# Systemic Lupus Erythematosus (SLE) Complicated by Human Immunodeficiency Virus (HIV): A Rare and Challenging Association

**DOI:** 10.7759/cureus.91928

**Published:** 2025-09-09

**Authors:** Satya Rijal, Aishwarya Joshi, Khanal P

**Affiliations:** 1 Internal Medicine, University of Michigan Health-Sparrow Hospital, East Lansing, USA

**Keywords:** antiviral therapy, complication, human immune deficiency virus, immunosuppressive agents, systemic lupus erythematosus, unique association

## Abstract

Systemic lupus erythematosus (SLE) is a chronic autoimmune disorder characterized by multi-organ inflammation, while human immunodeficiency virus (HIV) primarily compromises host defense by depleting CD4+ T lymphocytes. The pathogenesis of SLE involves dysregulated immunity with autoantibody and immune complex formation, whereas HIV-induced immunosuppression paradoxically predisposes individuals to autoimmune phenomena. The complex interplay between HIV and SLE remains poorly understood, highlighting the importance of accurate differentiation and individualized management in patients affected by both conditions and collaborative management across specialties.

We report the case of a 41-year-old male with a three-year history of SLE and suspected lupus nephritis, who had declined a renal biopsy. He later presented with clinical features suggestive of HIV infection. The case underscores the challenges of balancing immunosuppressive therapy in the setting of concomitant autoimmune disease and viral immunodeficiency. The patient was successfully managed with the antiretroviral regimen of dolutegravir and lamivudine, while continuing hydroxychloroquine and corticosteroids. Rituximab was discontinued due to safety concerns and potential drug interactions, considering the benefits of hydroxychloroquine and steroids in managing specific conditions.

## Introduction

The 2012 Systemic Lupus International Collaborating Clinics (SLICC) criteria require the presence of at least four of seventeen items, including at least one clinical and one immunological finding, to establish a diagnosis [1,2]. Despite advances in classification, diagnosing SLE remains challenging when overlapping conditions complicate the clinical picture [1-3]. Human immunodeficiency virus (HIV) infection further complicates the diagnosis of SLE. HIV primarily leads to progressive immunosuppression, increased susceptibility to infections, and paradoxical predisposition to autoimmunity [4,5]. Notably, HIV infection can induce autoantibodies such as ANA and anti-dsDNA, creating potential for false-positive serological tests that mimic SLE [5,6]. This overlap poses significant barriers to accurate diagnosis and timely management [5,6].

Epidemiologic reports suggest that SLE and HIV co-occurrence is uncommon but demonstrates distinct demographic patterns. Women, particularly African American women, appear disproportionately affected, consistent with the general epidemiology of lupus [4-6]. The sequence of diagnoses also varies, with pediatric patients more often presenting with HIV first, while adults are typically diagnosed with SLE in their late twenties or thirties [4-6].

The clinical course of patients with both conditions is complex. Shared features such as joint pain, skin rashes, and renal involvement complicate differentiation between lupus activity and HIV-related pathology. Furthermore, initiation of antiretroviral therapy (ART) may provoke immune reconstitution inflammatory syndrome (IRIS), which can exacerbate lupus manifestations [7,8]. These challenges necessitate a multidisciplinary approach to care [8].

Management requires balancing immunosuppression to control lupus activity with the preservation of immune function to prevent opportunistic infections. Drug interactions between ART and immunosuppressive agents further complicate therapy. While agents such as cyclophosphamide may require dose modification or avoidance due to infection risk, hydroxychloroquine has demonstrated both safety and possible antiviral benefit in HIV-positive patients [9-11].

## Case presentation

We present a clinical case involving a 41-year-old male patient who attended the rheumatology clinic with symptoms indicative of SLE. These symptoms included a malar rash and polyarticular inflammatory arthritis affecting the bilateral elbows, ankles, hands, and wrists for six months. The patient described his joint pain as coming on suddenly and being quite severe, with both sides affected symmetrically. He also reported morning stiffness that lasted over two hours. Over-the-counter pain medicines did not help much with the pain. The patient also exhibited alopecia associated with oral ulcers that would resolve independently. Aside from that, he denied any family history of autoimmune conditions.

The physical examination revealed tenderness in the bilateral wrists, elbows, metacarpophalangeal joints, and interphalangeal joints. No abnormalities were observed in the other joints. The photosensitivity rash was prominent on the cheeks.

Laboratory investigations revealed a hemoglobin level of 11.5 g/dL, a lymphocyte count of 0.6×10^9^/L, a platelet count of 170000/mcL, an elevated erythrocyte sedimentation rate (ESR) of 80 mm/h, and C-Reactive Protein (CRP) of 40 mg/dL. All other baseline investigations were within normal limits. The patient underwent an antinuclear antibody (ANA) profile, which demonstrated elevated titers of ANA (1:640) and positive anti-Smith antibodies (8.96 U/mL). Further examination revealed a positive result for anti-double-stranded DNA (anti-dsDNA) antibodies and decreased levels of C3 and C4. In addition, he had 3+ proteinuria on urine dipstick testing and an elevated creatinine level of 2.2 mg/dL.

The lupus anticoagulant (LA), anticardiolipin antibodies (ACL), and anti-beta-2 glycoprotein I (anti-B2GPI) antibodies, which are recognized as the markers for antiphospholipid syndrome (APS), were found to be negative (Table 1).

**Table 1:** Initial visit laboratory values and clinical data ESR: erythrocyte sedimentation rate; CRP: C-Reactive Protein; ANA: antinuclear antibody; dsDNA: double-stranded DNA; LA: lupus anticoagulant; ACL: anticardiolipin antibodies; B2GPI: beta-2 glycoprotein I

Parameter	Value/result	Normal range	Units
Hemoglobin	11.5	12-16	g/dL
Lymphocyte count	0.6	1.0-4.8	10⁹/L
Platelet count	170000	150000-450000	/mcL
ESR	80	0-20	mm/h
CRP	40	<30	mg/dL
ANA	1:640	Negative	Titer
Anti-Smith antibodies	8.96	Negative	U/mL
Anti-dsDNA antibodies	Positive	Negative	-
C3	Decreased	90-180	mg/dL
C4	Decreased	10-40	mg/dL
Urine protein dipstick	3+	Negative	-
Serum creatinine	2.2	0.6-1.2	mg/dL
LA	Negative	Negative	-
ACL	Negative	Negative	-
Anti-B2GPI	Negative	Negative	-

The patient received a diagnosis of SLE according to the SLICC criteria [3]. The possibility of renal involvement from SLE was discussed with the patient, including the potential need for a nephrology referral. However, the patient declined a nephrology referral at that time, stating a preference to proceed with medication management first and to consider referral if there is no clinical improvement. Subsequently, the patient was sent home with a prescription for prednisolone and hydroxychloroquine. He attended his follow-up appointment four months later, during which a complete blood count (CBC) revealed a consistent lymphocyte count of 0.6×10^9^, platelets measured at 170000 per microliter, creatinine of 1.9 mg/dL, an ESR of 80 mm/h, and CRP of 40 mg/dL, revealing the active SLE. Consequently, the same treatment regimen was upheld.

The patient demonstrated improvement in pain management and generally reported "positive progress" during a six-month follow-up for systemic lupus erythematosus, reflecting clinical improvement in symptoms, quality of life, or overall well-being. Objective measures of disease activity, such as laboratory tests including C3/C4 complement levels, anti-dsDNA antibodies, and ESR/CRP, were obtained. The lymphocyte count was 0.6 (10^9^/L), below the normal range of 1-4.8 (10^9^/L). ESR remained elevated at 80 mm/h (normal: 0-20 mm/h), and CRP was 40 mg/dL (normal: 0-10 mg/dL). C3 was 80 mg/dL (normal 90-80 mg/dL), C4 was 5 mg/dL (normal 10-40 mg/dL), and anti-dsDNA antibodies were positive. Creatinine was elevated at 2.2 mg/dL (normal: 0.6-1.2 mg/dL) (Table 2).

**Table 2:** Six-month follow-up laboratory values ESR: erythrocyte sedimentation rate; CRP: C-Reactive Protein; HIV: human immunodeficiency virus

Parameter	Value	Normal range	Units
Hemoglobin	10.5	12-16	g/dL
Lymphocyte count	0.6	1.0-4.8	10⁹/L
Platelet count	170000	150000-450000	/mcL
ESR	80	0-20	mm/h
CRP	40	<10	mg/dL
HIV-1 antigens and antibodies	Negative	Negative	

Clinical examination revealed no joint swelling, rashes, or organ involvement. Despite the patient's subjective improvement, persistent lymphopenia, ongoing renal involvement, and elevated ESR/CRP indicated active SLE disease with kidney involvement, warranting continued vigilance and possible further adjustments to treatment. The patient remained on steroids and hydroxychloroquine, with added cyclophosphamide to suppress disease activity and prevent relapses with reduced risk of severe manifestations. Upon discussion, a referral was made to a nephrologist for a kidney biopsy, but the patient refused a kidney biopsy, and the nephrologist indicated that dialysis was unnecessary.

The patient had follow-up visits in the rheumatology clinic every three months to monitor disease progression. However, at the six-month follow-up after starting cyclophosphamide, side effects of infertility were noted. Due to persistent disease activity and renal involvement, therapy was switched to rituximab, and a maintenance dose of steroids and hydroxychloroquine was continued. However, three years following the diagnosis of SLE, he presented to the hospital exhibiting oral thrush, diarrhea, significant weight loss, and lymphadenopathy. The diarrhea was characterized as watery, copious, and malodorous, with a frequency of eight to nine episodes per day, accompanied by nausea, abdominal cramps, anorexia, and a low-grade fever, alongside a weight reduction of ten pounds.

The patient appeared to be in stable condition. The physical exam showed that the patient had oropharyngeal thrush. The abdomen was soft, not swollen, and exhibited no tenderness upon palpation. Bowel sounds were high-pitched, but there was no sign of shifting dullness, splenomegaly, hepatomegaly, or swollen lymph nodes. Checks of the lungs, heart, and nervous system all came back normal. Blood tests revealed a hemoglobin level of 10.5 g/dL, lymphocytes at 0.6×10^9^/L, and an 80 mm/h ESR. Serum creatinine was 2.2 mg/dL, and urea was 40. The urine dipstick revealed 1-2 leukocytes and a +++ protein level. A 24-hour urine protein measurement showed 3 grams per day. A total bilirubin level of 0.52 mg/dL and serum glutamate pyruvate transaminase (SGPT) of 49 U/L. Serum sodium was 134 mmol/L, potassium at 3 mmol/L, chloride at 101 mmol/L, and bicarbonate at 20 mmol/L. The stool test showed mucus and about 10 white blood cells. We also did a stool culture, which came back negative for bacteria. Based on our observations, there was a suspicion of HIV. Further testing confirmed the presence of reactive HIV-1 antigens and antibodies. The workup showed that the patient had poorly controlled HIV, evidenced by a CD4 count of 100 cells/µL and 8.8×10^5^ copies/mL (Table 3).

**Table 3:** Hospitalization after three years with laboratory values ESR: erythrocyte sedimentation rate; SGPT: serum glutamate pyruvate transaminase; WBC: white blood cell; HIV: human immunodeficiency virus

Parameter	Value	Normal range	Units
Hemoglobin	10.5	12-16	g/dL
Lymphocyte count	0.6	1.0-4.8	10⁹/L
Platelet count	170000	150000-450000	/mcL
ESR	80	0-20	mm/h
Serum creatinine	2.2	0.6-1.2	mg/dL
Urea	40	7-20	mg/dL
Urine dipstick protein	3+	Negative	-
Urine leukocytes dipstick	1-2	Negative	-
24-hour urine protein	3	<0.15	grams/day
Total bilirubin	0.52	0.2-1.2	mg/dL
SGPT (ALT)	49	7-56	U/L
Serum sodium	134	135-145	mmol/L
Serum potassium	3	3.5-5.0	mmol/L
Serum chloride	101	98-107	mmol/L
Bicarbonate	20	22-29	mmol/L
Stool mucus	Present	Absent	-
Stool WBCs	~10	Absent	/HPF
Stool culture	Negative	Negative	-
HIV-1 antigens and antibodies	Reactive	Negative	-
CD4 count	100	500-1500	cells/µL
HIV viral load	8.8x10^5^	Undetectable	copies/mL

Treatment for SLE was stopped immediately, and the patient was referred to Infectious Diseases for further management. The patient was advised to follow up with an outpatient nephrologist again for the renal biopsy. The patient was initiated on dolutegravir and lamivudine by an infectious disease specialist and was advised to continue therapy with hydroxychloroquine and corticosteroids, with careful monitoring of his HIV status and immunosuppressive treatment. He was taken off rituximab due to concerns about its side effect safety profile in immunocompromised individuals and potential drug interactions, as well as the known benefits of hydroxychloroquine and steroids in managing specific conditions, even in the context of HIV.

Three months after starting ART, the HIV viral load dropped below detectable levels. The anti-ds-DNA antibody and complement levels normalized, decreasing SLE disease activity. The patient's joint symptoms fully resolved. Laboratory tests showed improved inflammatory markers, and daily urinary protein levels were reduced to 0.11 grams (Table 4). The patient had no complications from ART.

**Table 4:** Post-ART follow-up laboratory values ART: antiretroviral therapy; HIV: human immunodeficiency virus

Parameter	Value/result	Normal range	Units	Improvement
HIV viral load	Undetectable	Undetectable	copies/mL	Significant reduction from 8.8×10^5^
Anti-dsDNA antibodies	Normalized	Negative	-	Normalized from positive
C3	Normalized	90-180	mg/dL	Normalized from decreased
C4	Normalized	10-40	mg/dL	Normalized from decreased
Joint symptoms	Fully resolved	No symptoms	-	Resolved
Inflammatory markers	Improved	Variable	-	Indication of decreased disease activity
24-hour urine protein	0.11	<0.15	grams/day	Significant reduction from 3 grams/day

## Discussion

The coexistence of HIV infection and SLE presents a unique clinical challenge, as overlapping immunologic features complicate diagnosis and management. Several case series have shown that patients with both HIV and SLE can achieve clinical stability through a combination of ART and standard lupus treatments, though early onset of either condition is associated with higher mortality [7].

Interestingly, initial lupus manifestations may appear attenuated following HIV infection due to virus-induced immunosuppression. However, the initiation of ART and subsequent immune recovery can unmask or exacerbate SLE activity through immune reconstitution [8,11]. Some studies suggest that SLE may even confer partial protection against HIV infection. Cross-reactivity has been reported between autoantibodies in SLE and HIV proteins, including gp120 and gp41, and elevated cytokines such as IL-16 and IL-10 in SLE may inhibit viral replication. Additionally, hydroxychloroquine, a mainstay of lupus therapy, has been shown to interfere with HIV-1 transcription, highlighting potential therapeutic overlap [8-10].

Diagnosis in HIV-positive patients requires careful evaluation due to overlapping features. While ANA may be present in both conditions, anti-dsDNA and other SLE-specific autoantibodies (anti-Smith, anti-Ro/SSA) are generally absent in HIV, providing a distinguishing marker [9-11]. Confirmation of HIV infection relies on serologic testing, including fourth-generation immunoassays, Western blot, and HIV RNA PCR, whereas SLE activity is monitored by complement levels, anti-dsDNA titers, and clinical manifestations [10,11]. Our patient presented initially with classic SLE manifestations, but later was diagnosed with HIV infection almost three years into its disease course. Laboratory evaluation revealed elevated anti-dsDNA antibodies, low complement levels, proteinuria, and active joint involvement, while initially diagnosed with SLE. HIV was confirmed during hospitalization after three years with detectable viral RNA. Differential considerations included drug-induced lupus, opportunistic infections presenting with autoimmune-like features, and IRIS after ART initiation [11].

The management strategy prioritized both viral suppression and control of autoimmune activity. Initiation of ART was associated with rapid immunologic recovery, evidenced by an undetectable HIV viral load within three months. Standard SLE therapies, including hydroxychloroquine, were continued, taking advantage of its additional antiviral properties, while avoiding high-dose immunosuppression that could exacerbate viral replication [9-11]. Over three months, follow-up evaluations showed undetectable HIV viral load, normalization of anti-dsDNA and complement levels, reduction in proteinuria, and improvement in joint symptoms, demonstrating concurrent control of both diseases. Importantly, ART was well-tolerated with no adverse effects, underscoring its dual therapeutic activity for both HIV and SLE. 

Our patient tolerated ART and lupus-directed therapy without complications, highlighting the safety of carefully coordinated treatment in coexisting HIV and SLE. This case also illustrates an important clinical phenomenon: immune reconstitution with ART can unmask or modulate autoimmune manifestations, emphasizing the need for vigilant monitoring of CD4 counts, viral load, and lupus disease activity. The observed improvement suggests that, in selected patients, ART may not only control HIV but also indirectly mitigate SLE flares by restoring immune homeostasis [12,13].

## Conclusions

This case report demonstrates that early and coordinated management of coexisting HIV infection and SLE can result in rapid and sustained clinical and immunological improvement. Accurate differentiation between HIV-associated immune phenomena and true autoimmune activity is essential for proper diagnosis and treatment planning. Our patient’s course highlights the dual benefit of ART, which not only suppresses viral replication but also modulates autoimmune activity, enabling the safe and effective use of lupus-directed therapies. Vigilant monitoring, individualized treatment adjustments, and a multidisciplinary approach are key to optimizing outcomes in patients with concurrent HIV and SLE, thereby reducing morbidity and improving long-term prognosis.
